# A technique for measuring non-structural carbohydrate reserves in flag leaves of paddy rice using Fourier transform infrared spectroscopy (FTIR)

**DOI:** 10.1186/s13007-025-01444-y

**Published:** 2025-10-31

**Authors:** Kharla Mendez, M. Arlene Adviento-Borbe, Cherryl Quiñones, Wenceslao Larazo, Brian Ottis, Argelia Lorence, Harkamal Walia

**Affiliations:** 1 Mississippi Water Resources Research Institute (WRRI), Mississippi State University, Mississippi State, Starkville, MS USA; 2 Delta Water Management Research Unit, Department of Agriculture (USDA) Agricultural Resources Services (ARS), Jonesboro, AR USA; 3 Biological and Agricultural Engineering, University of Arkansas, Fayetteville, AR USA; 4 Arkansas Biosciences Institute, Arkansas State University, Jonesboro, AR USA; 5 US Marketing and Product Management, RiceTec, Inc., Houston, TX USA; 6 Department of Chemistry and Physics, Arkansas State University, Jonesboro, AR USA; 7 Department of Agronomy and Horticulture, University of Nebraska-Lincoln, Lincoln, NE USA

**Keywords:** Non-structural carbohydrates, Rice, Leaves, FTIR, High nighttime temperature

## Abstract

The application of Fourier transform infrared (FTIR) spectroscopy for non-structural carbohydrates (NSC) prediction as a tool for pre-breeding screening has immense potential but remains to be unexplored, because of technical challenges associated with these measurements. This study investigated the potential of employing FTIR spectroscopy as a high-throughput tool for forecasting NSC content, including total soluble sugar (TSS) and starch content, of 30 rice accessions from the Rice Diversity Panel 1 (RDP1) germplasm and RiceTec hybrids grown in 2019 (320 genotypes) and 2020 cropping (312 genotypes). Partial Least Squares (PLS) regression analysis was used to construct predictive models to estimate NSC content in flag leaves and stem of rice exposed to elevated and ambient nighttime air temperature during the flowering stage of rice. The TSS model exhibited a coefficient of determination (R^2^) value of 0.63 and root mean square error of prediction (RMSEP) values of 3.62 mg g^− 1^. Notably, the NSC model demonstrated a superior metric performance, with R^2^ = 0.66 and RMSEP of 5.58 mg g^− 1^. The predictive model created in this research effectively measured the NSC composition present in the flag leaves of rice. Expanding the sample size and incorporating additional principal components may enhance the model’s predictive accuracy. The FTIR technique can produce fast accurate results and resolve the high analytical costs. Overall, the use of FTIR in conjunction with PLS regression analysis provides a potential tool to advance our understanding of various rice genotypes, particularly concerning their ability to withstand abiotic stress such as HNT.

**Supplementary Information:**

The online version contains supplementary material available at 10.1186/s13007-025-01444-y.

## Background

The concentration of non-structural carbohydrates (NSC) in grass stems plays a crucial role in yield and stress tolerance [1]. In paddy rice, 30–40% of the grain mass is stored in stem reserves before grain formation under normal conditions [2]. Unlike other grasses, rice exhibits distinct carbohydrate partitioning dynamics, storing sugar in leaves and both starch and sugar in stems [3]. A temporary sink is essential, as up to 28% of the final stored carbohydrates are translocated from stems to grains [4].

Abiotic factors—including high nighttime temperature (HNT) stress, play a significant role in rice carbohydrates partitioning. Although nighttime temperatures are generally lower than daytime temperatures, recent studies have observed a rapid increase in nighttime air temperatures [5, 6]. Numerous publications have documented the negative impact of HNT stress on rice grain yield [5, 7–9] because HNT stress affects the carbohydrate content of rice that results in impaired rice growth, stress response and grain yield. The main driver of HNT-induced yield loss in rice is caused by night respiration with relation to tissue carbohydrates status [10]. HNT accelerates respiration, reducing nonstructural carbohydrates such as sucrose, starch, glucose, and fructose, particularly when respiration surpasses photosynthesis in rice [11–14]. HNT-tolerant rice with greater stem storage capacity, demonstrated improved translocation, reduced spikelet sterility, and stable 1000-grain weight [14]. A study on the HNT-susceptible rice variety Gharib found that yield reduction was linked to decreased NSC translocation after flowering, which also influenced the grain filling rate [15]. Although most of the studies highlighted the importance of NSC storage in stem [8, 16, 17], there are studies that indicated that HNT stress affects NSC dynamics in flag leaves. In rice, the flag leaf is the final leaf to develop on a mature flowering stem. Positioned at the top of the canopy, it intercepts more light than the lower leaves, enabling a higher photosynthetic capacity [18]. Moreover, the size and health of flag leaves has been strongly linked to rice yields, as they supply half of the photosynthetic products needed for grain development [19–21]. Research also suggests that HNT disrupts the normal day/night metabolic cycle of NSC accumulation in flag leaves, potentially reducing their ability to supply carbohydrates for grain filling [22]. Additionally, HNT has been linked to increased dark respiration in flag leaves, which may accelerate NSC depletion and contribute to yield loss [16].

Given the critical role of NSC in rice productivity and tolerance to HNT, infrared (IR) spectroscopy has proven instrumental in assessing NSC composition in plant tissues, including flag leaves. Specifically, IR enables rapid, non-destructive evaluation of biochemical components, facilitating a deeper understanding how HNT stress influences translocation and overall plant resilience [16, 17]. In addition to carbohydrates, IR spectroscopy proves to be useful in measuring nitrogen content [23, 24], protein content [25], lipid concentration [26, 27] and antioxidant level [28] in rice. The fundamental principle of NIR spectroscopy relies on the concept that biological samples contain organic and inorganic compounds with CH, OH and NH bonds, which undergo stretching and bending when excited by electromagnetic radiation in the near-infrared region (wavelength: 780 to 2,500 nm, wavenumber: 12,820.5 to 4,000 cm^− 1^) of the electromagnetic spectrum [29].

Fourier transform infrared (FTIR) spectroscopy has effectively been applied in the high throughput measurement of NSC in plant tissues and extracts. Marker bands of saccharides were established to show correlation with their structural features, despite having less distinct vibrational spectra when compared to lipids and proteins [30]. The effectiveness of FTIR was demonstrated through the development of predictive models with attenuated total reflectance (ATR) FTIR to measure non-structural carbohydrates in apple, peach, and orange fruit juices [31]. The statistical analysis presented in the paper of Leopold et al. [31], particularly focusing on the root mean square error of prediction (RMSEP), indicated that the first derivative spectra transformation led to the smallest prediction errors of 0.070 g of glucose per 100 mL. A different study also effectively created a multi-year predictive model for non-structural carbohydrates of different onion varieties through partial least square (PLS) regression [32]. Performance statistics of 100 models demonstrated best prediction result with fructose (RMSEP = 1.355, R^2^ = 0.90) and glucose (RMSEP = 2.986, R^2^ = 0.84) but not with sucrose (RMSEP = 2.076, R^2^ = 0.94) datasets due to limited variability in concentration ranges [32].

FTIR-based screening methodology to assess different levels of NSC has been implemented on plant species belonging to the grass family. The prediction of soluble sugars of a large population of *Sorghum bicolor* was rapidly assessed with 4% margin of error using PLS modelling via Unscrambler X [33]. A parallel approach was employed to evaluate the range of stem NSC levels of rice accessions from multi-parent advanced generation inter-cross developed for heat tolerance (MAGIC^heat^) as well as chosen rice breeding lines recognized for their ability to withstand high temperatures [16]. In this research, the PLS models were developed using an R statistical programming language and yielded RMSEP values of 1.559, 1.437 and 0.573 for total NSC, soluble sugar and starch, respectively. The above referenced studies demonstrated the efficiency of FTIR spectroscopy and the application of statistical methods in the field of chemometrics, particularly PLS analysis.

Despite ongoing advancements in the application of FTIR spectroscopy for NSC prediction, its utilization in pre-breeding screening remains to be relatively constrained. In this study, we explored the use of FTIR spectroscopy as a high-throughput tool to predict the NSC content using rice plants parts from selected rice accessions grown under ambient and elevated HNT during flowering stage. The study’s specific objectives involved (1) assessing the total soluble sugar and starch content in both leaves and stems using conventional NSC analysis and (2) developing and verifying an FTIR model using datasets derived from conventional methods with minimal error estimates.

## Materials and methods

### Source of plant samples

#### Field experiment

Plant biomass samples used in model development were obtained from the field experiments study on yield response of various rice selections from worldwide origins under heat stress. Two field experiments were conducted in 2019 and 2020 at the RiceTec Research Experimental Station in Harrisburg, AR, USA. The field trials were arranged in a randomized complete block design (RCDB) with three replications. Main treatment was air temperature (ambient vs. heated) and sub-treatment was 320 rice selections from Rice Diversity Panel 1 (RDP1) [34, 35] and hybrid rice cultivars (RiceTec, Inc, TX, USA). The RDP1 panel was selected due to its broad genetic diversity, which captures the variability across diverse geographic regions and ecological settings [36]. In each replication, rice varieties were grouped by height—short, medium, and tall—then randomized to reduce shading and maintain uniform solar radiation exposure. Rice plants were grown under continuously flooded irrigation and urea fertilizer was surface applied before permanent flooding at 120 kg N ha^− 1^. Weed control was achieved using herbicide applications recommended for these systems.

At physiological maturity, rice plants were manually harvested from each replicate plot. Panicles were manually threshed and separated the filled and unfilled grains. The filled grains were air-dried to 14% moisture content and dried grains were weighed to measure plot grain yield.

#### HNT stress treatment

For the air temperature treatment, three high tunnel greenhouses (9.14 m wide x 16.63 m long x 4.39 m tall) were used for the control or actual ambient nighttime air temperature and another three greenhouses were used for HNT at flowering stage. In the HNT greenhouses, the field-based infrastructure and cyber-physical system were implemented and effectively applied temperature stresses of 4.0 °C in 2019 and 3.94 °C in 2020 [37]. The variations in nighttime air temperatures within enclosed greenhouses can be linked to fluctuations in relative humidity. The greenhouses were positioned over the experimental rice field once 50% of the total population of the rice genotypes had reached the flowering stage. The plants were subjected to HNT stress from 18:00 to 05:00 for two weeks, with all rice varieties in the FTIR study experienced full HNT stress. Within each greenhouse, 320 rice genotypes were manually sown in a 60 cm long x 20 cm wide rows with a 20 cm distance between rows of rice and 7 cm between rice plants. A total of 40 seeds per variety were sown per replication. Five seeds were planted in each rice hill to achieve 100% plant emergence. In the 2020 experiment, 312 rice genotypes were planted due to limited availability of seeds and some rice selections did not grow under field conditions in Arkansas. The prolonged daylight conditions in Arkansas may have delayed or entirely prevented flowering of rice genotypes.

### Plant material

The plant materials used for model development were 12 genotypes selected based on their yield responses to ambient and HNT stress from the field experiments discussed above. The 12 plant materials were composed of four HNT-tolerant varieties namely Dourado Agulha, Keriting Tingii, NSF-TV 13 and NSF-TV 2, four HNT-sensitive varieties namely Binulawan, Kasalath, S4542A3-49B-2B12, and TOg 7178, and four hybrid varieties (RT7301, XP753, XP754, and XP760).

#### Sample collection and processing

Plant samples were collected at flowering and dough growth stages. Two main tillers were collected from the middle plot, with 15-day interval between sampling dates. The main tillers were divided into two tissue types: leaves and stems. To inhibit enzymatic activity, all samples were promptly microwaved for 60 s. Tissue samples were further dried using convection oven at 60 °C for 3 days, and individual oven-dry weights were recorded. The oven-dry weight served as a standardization measure for glucose content across the diverse biomass sample set.

#### Plant sample sub-sets for model development and data prediction

For the development of the FTIR model, this study used two distinct biomass sample sets. The first set (*n* = 565) was utilized for the development of the PLS regression model, whereas the second set (*n* = 60) was employed to validate the PLS regression model. Both sample sets were collected during the 2019 and 2020 cropping. Sample set 1 was used to develop Model 1 and 2, which was composed of leaf and stem tissue with 302 samples in 2019 (143 leaves + 159 stem) and 263 samples in 2020 (133 leaves + 130 stem) with a total of 565 plant samples. Sample count varied due to plant mortality and availability, as the same samples were used for wet chemistry analysis. Small tissue quantities were also influenced by constrained by resources, manpower, and the intricate field sampling methodology.

A second sample set was created for data prediction using Model 2. The sample set (sample set 2) consisted of samples collected in 2019 (*n* = 30) and 2020 (*n* = 30), which were selected based on the highest and lowest grain yield genotypes as observed in the field from each cropping year. Grain yield was selected to streamline the sample set for data prediction, as it is the primary indicator of rice tolerance and susceptibility to HNT stress [38–40]. Unlike sample set 1, this set used flag leaf tissues which were collected from the main tiller. Sample set 2 maintained a consistent size across both years, as they were easier to collect than main tillers, which required cutting from the plant base. Prior to FTIR analyses, all plant tissue samples were shocked-frozen in liquid nitrogen and stored at − 80 °C freezer to preserve the integrity of the samples.

#### Conventional method of non-structural carbohydrates analyses

Wet chemistry analysis was conducted on the first samples set to obtain the reference value for the FTIR model development. The dried tissue samples underwent pre-processing with the Eberbach cutting mill (Eberbach Corporation, Van Buren Charter Township, MI) to attain a particle size of 2 mm. Approximately 100 mg of dried ground samples were subjected to chemical analysis using an acid method [41]. The quantification of NSC involved summation of total soluble sugar (TSS) and starch. TSS extraction utilized 80% ethanol, while starch extraction involved 80% ethanol and perchloric acid at two different concentrations (9.2 N and 4.6 N). Colorimetric measurements of both TSS and starch content were carried out by adding the color-producing agent anthrone. A UV-1800 spectrophotometer (Shimadzu Scientific Instruments, Columbia, MD) was used for colorimetric analysis at 620 nm and 630 nm for TSS and starch, respectively. Anhydrous glucose served as the standard, with concentrations ranging from 0 to 600 ppm.

### Hyperspectral imaging system and model development

#### FTIR sample preparation

**Fig. 1:**
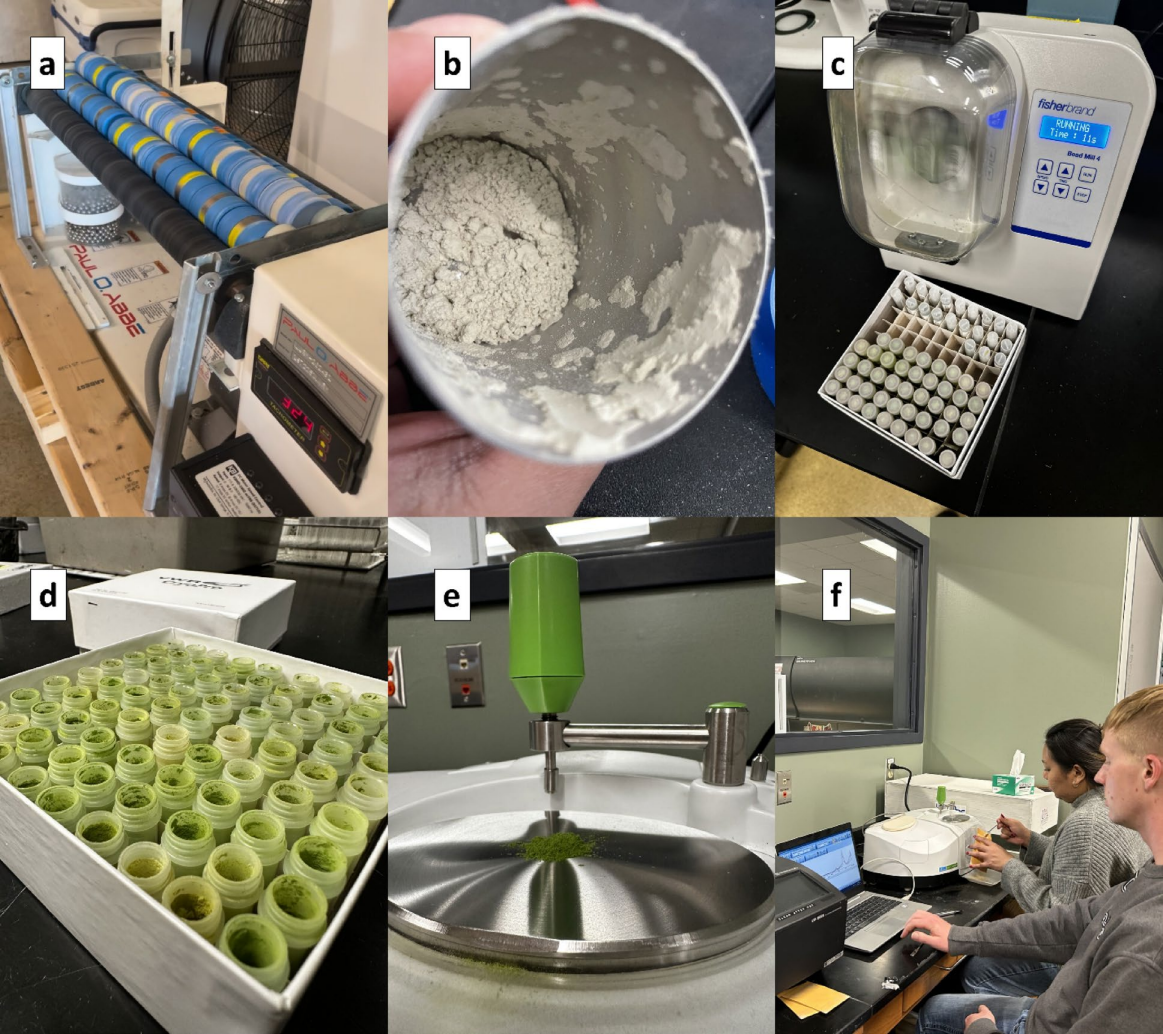
Images showcasing the materials, equipment, and procedures used during the experiment. **a** Jar rolling mill employed for grinding stem and leaf samples in set 1, **b** ground plant sample contained within a modified stainless steel core sampler, **c** bead mill homogenizer utilized for processing flag leaf sample in set 2, **d** finely ground flag leaf samples contained in a plastic bead mill tube, **e** ground sample placed on the holding area of the Spectrum Two FT-IR Spectrometer, and **f** configuration of the computer set-up for controlling the FT-IR spectrometer and acquiring spectral data

Two methods were utilized for FTIR sample preparation due to the condition of the samples (Fig. 1). Sample set 1 composed of 2 mm sized tissues were finely ground using a jar rolling mill (Paul O. Abbé, Wood Dale, IL) for 3 to 7 days at 366 RPM, with sample weights ranging between 1 and 15 g. For sample set 2, flag leaves were immediately microwaved for 60 s and oven-dried for 3 days at 60 °C. Due to the small sample size, flag leaves were ground using a bead mill homogenizer (Fisherbrand™, Waltham, MA) at 375 RPM for 2 min, with significantly lower sample weight of approximately 200 mg. Although particle size was not measured, all samples were uniformly powdered after grinding. Both sample sets were dried at 60 °C for 12 h before spectra scanning to eliminate excess water content.

#### Scanning of tissue sample

Plant tissue samples from sample set 1 and 2 were used to quantify the FTIR spectrum using a Spectrum Two FT-IR Spectrometer (PerkinElmer, Inc., Waltham, MA) equipped with Spectrum IR software for spectral data acquisition (PerkinElmer, Inc., Waltham, MA). Infrared radiation wavelength was manually set at 400 to 4,000 cm^− 1^. Automatic baseline correction was applied using the Spectrum IR application installed on the spectrometer. Backgrounding was executed in the beginning of each run to ensure high quality and accuracy of the collected spectral data. Each sample was scanned four times with a total scan time of two minutes.

#### Development of partial least square (PLS) regression model

**Fig. 2:**
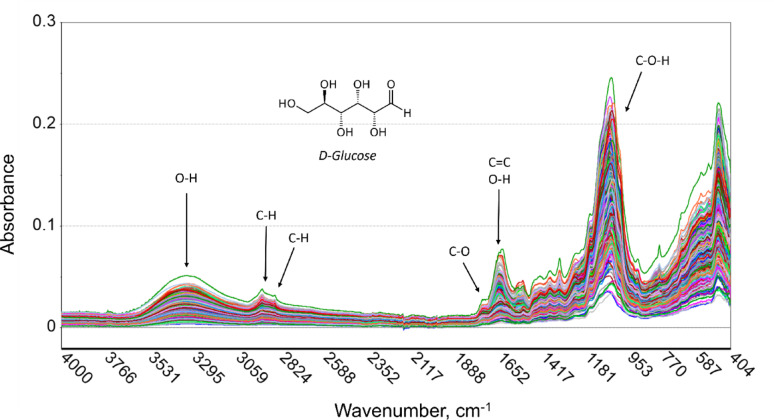
Assignment of main bands in original FTIR spectra of 565 rice stem and leaf tissue samples

The Unscrambler X version 10.5.1 software (Camo Analytics, Oslo, Norway) was used to perform multivariate data analysis. A total of 565 samples were pooled to fit the PLS model (Fig. 2). Spectral datasets for each plant sample were transformed using four data preprocessing steps: (1) Smoothing Savitzky-Golay Transform, (2) Baseline Transform, (3) 2nd Derivative Savitzky-Golay Transform, and (4) Standard Normal Variate Transform.

Initial model development was also performed using Unscrambler X. The number of latent variables or factors were determined. The factors were derived by using the covariance between the predictor (X) and response (Y) matrix, and by capturing the largest possible variance in X that effectively correlates with and explains Y.

For Model 1, the 565 tissue samples (leaf and stem) were divided into training (*n* = 465) and test (*n* = 100) sets by the Kennard-Stone algorithm. A test set of 100 samples was selected based on an 80:20 ratio, consistent with chemometric analysis of rice [42]. Model 2 was constructed exclusively from 276 leaf tissue samples to enhance the PLS model, with the dataset further partitioned into a training set (*n* = 219) and a test set (*n* = 57). Due to the limited dataset size, a larger training set was prioritized to improve pattern recognition and predictive accuracy. The utilization of the test set for external validation was crucial in the evaluation of the model’s performance, prevention of overfitting, and verification of the model’s capability to make accurate predictions of new or unseen data. The relationship between spectral data, which consists of 3600 data points, and wet chemistry data (for TSS, starch and NSC) was analyzed using PLS regression model following Kernel algorithm. To estimate the likely performance of the predictive model, cross-validation was used following a random method with 20 segments. To ensure an unbiased model evaluation, the test set was predicted by the developed PLS models. The test set spectra received the same pretreatment processes performed to the training set.

### Data analysis

Descriptive statistics and data visualization were performed using analysis package of SigmaPlot version 14.0 (Systat Software Inc., San Jose, CA). Reference and predicted datasets were compared and summarized using the following parameters: mean, confidence of interval of the mean, standard deviation, standard error, range, maximum value, minimum value and median.

Coefficient of determination (R^2^), root mean square error (RMSE), residual prediction deviation (RPD), and mean absolute percentage error (MAPE) were used to evaluate the predictive models. The performance metrics were calculated as follows:

1 $${R}^{2}=1-\left(\frac{{\sum\:}_{i=1}^{n}{({Gluc}_{i}^{O}-{Gluc}_{i}^{P})}^{2}\:\:\:\:\:}{{\sum\:}_{i=1}^{n}{({Gluc}_{i}^{O}-{\stackrel{-}{Gluc}}_{i}^{O})}^{2}\:}\right)\:\:\:\:\:\:\:\:\:\:\:\:\:\:\:\:\:\:\:\:\:\:\:\:\:\:\:\:\:\:\:\:\:\:\:\:\:\:\:\:\:\:$$

2 $$RMSE=\sqrt{\frac{{\sum\:}_{i=1}^{n}{({Gluc}_{i}^{O}-{Gluc}_{i}^{P})}^{2}\:\:\:\:\:}{n\:}}\:\:\:\:\:\:\:\:\:\:\:\:\:\:\:\:\:\:\:\:\:\:\:\:\:\:\:\:\:\:\:\:\:\:\:\:\:\:\:\:\:\:\:\:$$

3 $$\:RPD=\:\frac{\sqrt{\frac{\frac{1}{n\:}{\sum\:}_{i=1}^{n}{\left({Gluc}_{i}^{O}-{\stackrel{-}{Gluc}}_{i}^{O}\right)}^{2}\:\:\:\:\:}{n\:}}}{RMSE}\:\:\:\:\:\:\:\:\:\:\:\:\:\:\:\:\:\:\:\:\:\:\:\:\:\:\:\:\:\:\:\:\:\:\:\:\:\:\:\:\:\:\:\:\:\:\:$$

4 $$\:MAPE=\frac{1}{n}\:\left({\sum\:}_{i=n}^{n}\left|\frac{{Gluc}_{i}^{O}-{Gluc}_{i}^{P}}{{Gluc}_{i}^{O}}\right|\right){\:\times100\%}\:\:\:\:\:\:\:\:\:\:\:\:\:\:\:\:\:\:\:\:\:\:\:\:\:\:\:\:\:\:\:\:\:\:$$

where *n* is the total number of data points, $\:{Gluc}_{i}^{O}$ is the reference glucose content of the *i*th sample measured through wet chemistry, $\:{\overline{Gluc}}_{i}^{O}$ is the mean of the reference glucose contents, and $\:{Gluc}_{i}^{P}$ is the predicted glucose content computed using the PLS model. R^2^ provided information on the goodness of fit of the PLS regression models to the reference TSS, starch and NSC values. RMSE was computed to evaluate the accuracy of the calibration and validation models. RPD functioned as an indicator for assessing the predictive performance of the models by considering both prediction error and the variation of observed data. Similarly, MAPE is defined as a performance metric that measures accuracy with units of a percentage. MAPE was included as an extra measurement to offer a more well-rounded evaluation and second level metric of the effectiveness of PLS regression models.

## Results and discussion

### PLS regression models for TSS, starch and NSC contents

Actual concentrations of TSS, starch and NSC in stem and leaf samples of rice were chemically analyzed and ranged from 3.81 to 140.87 mg g^− 1^, 0.60–103.56 mg g^− 1^, and 6.52–195.37 mg g^− 1^, respectively. Highest mean glucose contents were measured in NSC and lowest in starch (Table 1). Results from these analyses were used to validate the calibration model for FTIR measurements.

**Fig. 3:**
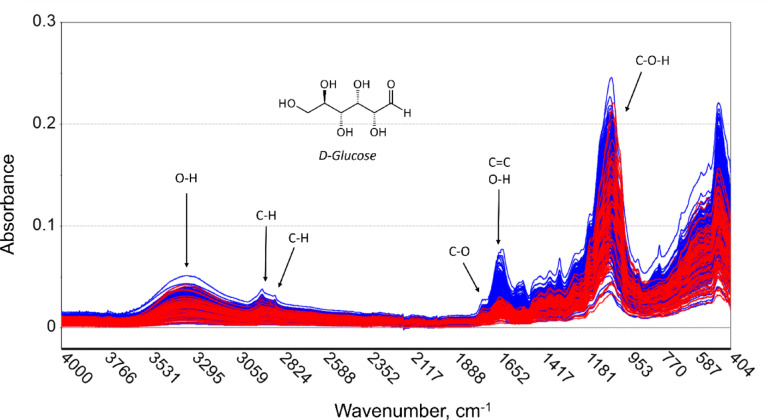
Original spectra grouped into plants parts. Red line represents stem samples and blue line represents leaf samples

A similar pattern of absorbance peaks was observed for both stem and leaves samples using the initial ATR-FTIR analysis with the spectra of stem samples being higher at wavenumbers 1045, 1637, 1739, 2922 and 3334 cm^− 1^ (Fig. 3). These prominent absorption bands were closely assigned to –C–H, –C=O and –C–O–H groups of carbohydrates, including fructose and glucose [27, 43]. Peaks of absorbance were caused by –OH stretching and C–H stretching in cellulose, C=C stretching in lignin, and overlapping of carbohydrates and lignin functional groups [44].

To enhance the signal and minimize noise, the original spectra were refined specifically in the regions between 3,642 and 2,925 and 1,850 to 400 cm^− 1^ (Figure S1a). Following the transformation, the peak of interest became more distinct after the post-transformation processes, specifically in wavenumbers of 1,200 to 1,600 cm^− 1^ (Figure S1b).

**Table 1:** Descriptive statistics of PLS model validation sample group (*n* = 100)

Descriptive statistics	TSS, mg g^− 1^		Starch, mg g^− 1^		NSC, mg g^− 1^	
	Reference	Predicted	Reference	Predicted	Reference	Predicted
Mean	37.55	32.71	17.75	19.19	55.30	50.61
Std. dev.	32.72	22.15	24.60	25.17	54.57	46.75
Std. error	3.27	2.22	2.46	2.52	5.46	4.68
Range	137.05	75.63	102.97	88.64	188.85	146.54
Max	140.87	81.87	103.56	84.50	195.37	151.83
Min	3.81	6.24	0.60	-4.15	6.52	5.30
Median	19.67	20.85	1.93	4.89	21.30	23.28

**Fig. 4:**
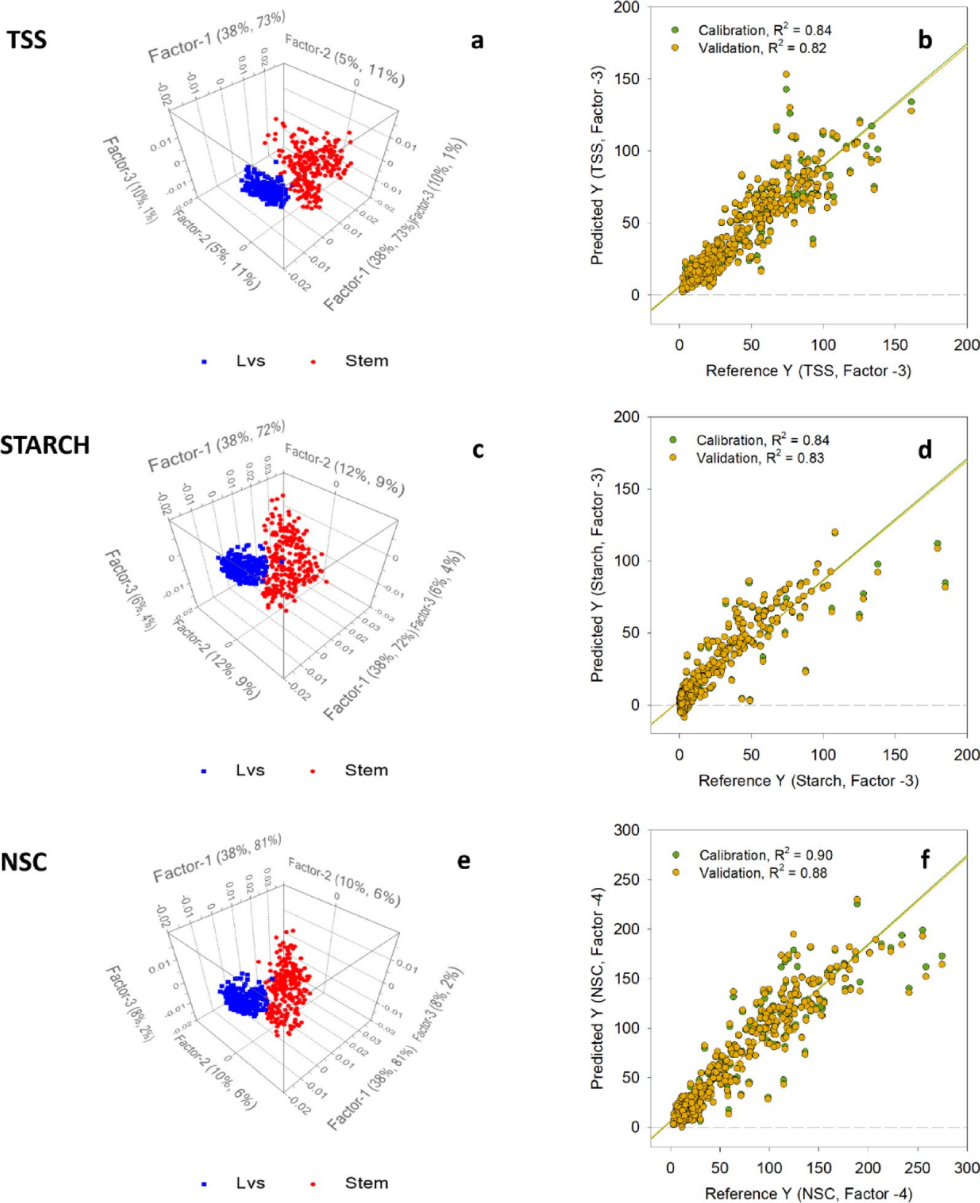
PLS scores categorized by plant parts and regression analysis reference vs. predicted concentrations. PLS scores were calculated for **a** TSS, **c** Starch, and **e** NSC concentrations in leaves and stems. Lvs denotes leaves. The regression analysis was conducted by individually calculating the reference and predicted concentrations of **b** TSS, **d** Starch and **f** NSC. Reference Y values were derived through wet chemistry analysis, whereas predicted Y values were determined using the FTIR method

Among 15 factors considered in the PLS regression analysis, three to four factors were selected for TSS, starch and NSC models with the highest R^2^ values ranging from 0.78 to 0.92 and lowest RMSE (7.9–19.1) (Table 2). At these selected number of factors, the variances among the spectra data were more closely aligned for both the training and internal validation model (Figure S2a, S2b and S2c) and variations in the spectra responses were considerably predicted by the three models. Among the three models, the NSC models displayed the highest percentage variations. The fitness of the models for both training and internal validation models were demonstrated in Fig. 4b, d and f. Figure 4d indicated the limitation of the starch model in predicting values in the lower range, especially when compared to TSS and NSC models. The reason for this constraint is linked to the existence of reference starch values falling 6.5 mg g^− 1^ glucose standard, which were then predicted to be below 0 mg g^− 1^. The results we gathered were opposite of R^2^ values observed in a similar study conducted by Xu et al. [16], where in the best fitted PLS regression model were developed out of starch dataset (R^2^ = 0.97), followed by NSC (R^2^ = 0.93) and TSS datasets (R^2^ = 0.86). According to Xu et al. [16], the predictive model exclusively utilized samples derived from stem. That being a case, the homogeneity of the samples source could have affected the R^2^ values in this study.

**Table 2:** Total soluble sugar (TSS), starch and non-structural carbohydrates (NSC) PLS models evaluation metrics using stem and leaves tissues (*n* = 565)

Statistics	TSS	Starch	NSC
Principal components in models	3	3	4
R^2^ Training (*n* = 465)	0.84	0.84	0.90
R^2^ Internal Validation (*n* = 465)	0.83	0.83	0.88
R^2^ External Validation (*n* = 100)	0.78	0.90	0.92
RMSEC (Training)	12.3	11.1	17.6
RMSECV (Internal Validation)	13.0	11.6	19.1
RMSEP (External Validation)	15.2	7.9	15.3
RPD_C_ (Training)	2.5	2.5	3.1
RPD_CV_ (Internal Validation)	2.4	2.4	2.8
RPD_P_ (External Validation)	2.1	3.2	3.5
MAPE_C_ (Training)	16%	–	9%
MAPE_CV_ (Internal Validation)	17%	–	10%
MAPE_P_ (External Validation)	6%	–	9%

Further assessment of the spectra datasets showed distinctive patterns among low, moderate and high values when utilizing factors 3, 3, and 4 for TSS, starch, and NSC model, respectively (Figure S3a, S3b, and S3c). Clear segregation of latent variables was also observed when the samples were categorized based on the tissue type (Fig. 4a, c and e). The noticeable clustering of latent variables observed based on tissue type confirmed the previous observation that the spectral characteristics of leaf and stem tissues were distinct from one another. To date, no research has been identified that demonstrates variation in FTIR spectra among different parts of rice plants. However, a comparable study revealed distinct FTIR spectra for various tissue types of cassava. The divergence in spectra between cassava leaves and stem was associated with variations in lignin and hemicellulose content, particularly in the fingerprint region spanning 730 to 1,830 cm^− 1^ [45]. This region indicates abundance in C-O-C (methoxy compound) stretching and C=O (aromatic ring) functionalities. In an FTIR analysis using rice straw, Swantomo et al. [46] demonstrated that the signal observed in the range of 1,000 to 1,150 cm^− 1^ was clarified as arising from the presence of cellulose. In fact, it was associated with the existence of alkoxy group and β-glycosidic network between glucose units. Given scientific evidence, developing separate models for leaf and stem samples could likely result in a more precise model.

PLS regression models are commonly assessed using R^2^ and RMSE values because both metrics provide a comprehensive evaluation of the model’s performance. In our study, RDP and MAPE were included as additional statistical metric to gauge the performance and accuracy of the predictive models. Based on RMSE, the internal validation for all parameters had higher prediction error compared to the training model. RMSE of PLS regression models of TSS, starch and NSC based on internal validation were 13.0 mg g^− 1^, 11.6 mg g^− 1^ and 19.1 mg g^− 1^, respectively. RPD values for training and internal validation sets ranged from 2.4 to 3.1, which demonstrates that the PLS model has a moderate to excellent predictive values in other crops [47, 48]. Stronger prediction based on RPD was observed with NSC model. MAPE was computed exclusively for TSS and NSC models, as the starch model contained 0 values. MAPE values showed that training and internal validation predicted comparable errors for both TSS and NSC. The MAPE calculation also revealed that NSC model (MAPE_C_ = 9%, MAPE_CV_ = 10%) had lower prediction error compared to the TSS model (MAPE_C_ = 16%, MAPE_CV_ = 17%). The shift in error levels of RMSE, RPD and MAPE was likely due to NSC consisting of the combined total of TSS and starch, resulting in higher values.

### External validation of PLS regression models

The performance of the calibration model was further assessed using 100 data points outside of the training set (calibration dataset). Two-thirds of the external validation dataset belonged to the lower range of the regression model. The deviation values ranged from 6.4 to 26.0 mg g^− 1^ for TSS model, 6.0 to 22.7 mg g^− 1^ for starch model and 10.5 to 41.7 mg g^− 1^ for NSC model (Figure S4a, S4b, and S4c). The R^2^ of the external validation was highest in NSC model (R^2^ = 0.92) followed by starch (R^2^ = 0.90) and TSS (R^2^ = 0.78) models. The R^2^ values of the external validation of all parameters exceeded the values previously observed in training model and internal validation of starch and NSC models (Table 2). However, values on the uppermost range did not fit well on PLS regression models for TSS (Figure S4d) and starch (Figure S4e).

Samples with starch concentrations between 0 and 10 mg g⁻¹ were assigned negative predicted values, indicating that the starch model lacks accuracy when estimating low starch content. Conversely, all randomly selected values for the external validation displayed a perfect fit to the PLS regression line using the NSC model (Figure S4f).

RMSE values of TSS, starch and NSC external validation are 15.2 mg g^− 1^, 7.9 mg g^− 1^ and 15.3 mg g^− 1^, respectively. The predicted values using the external validation dataset had the lowest RMSE in starch models. When compared to computed RMSE values, MAPE value was lower in the external validation of TSS model (MAPE_P_ = 6%) than NSC content (MAPE_P_ = 9%). No values for MAPE_P_ were obtained for starch external validation due to the presence of negative values. The starch (RPD_P_ = 3.2) and NSC (RPD_P_ = 3.5) models exhibited better predictive accuracy compared to the TSS model based on RPD_P_, yet all models successfully captured variations within the dataset. As supported by statistical metrics, external validation of the spectral datasets for all three C models illustrated that the variation in spectral data can reliably predict the NSC and TSS contents better than starch content.

**Fig. 5:**
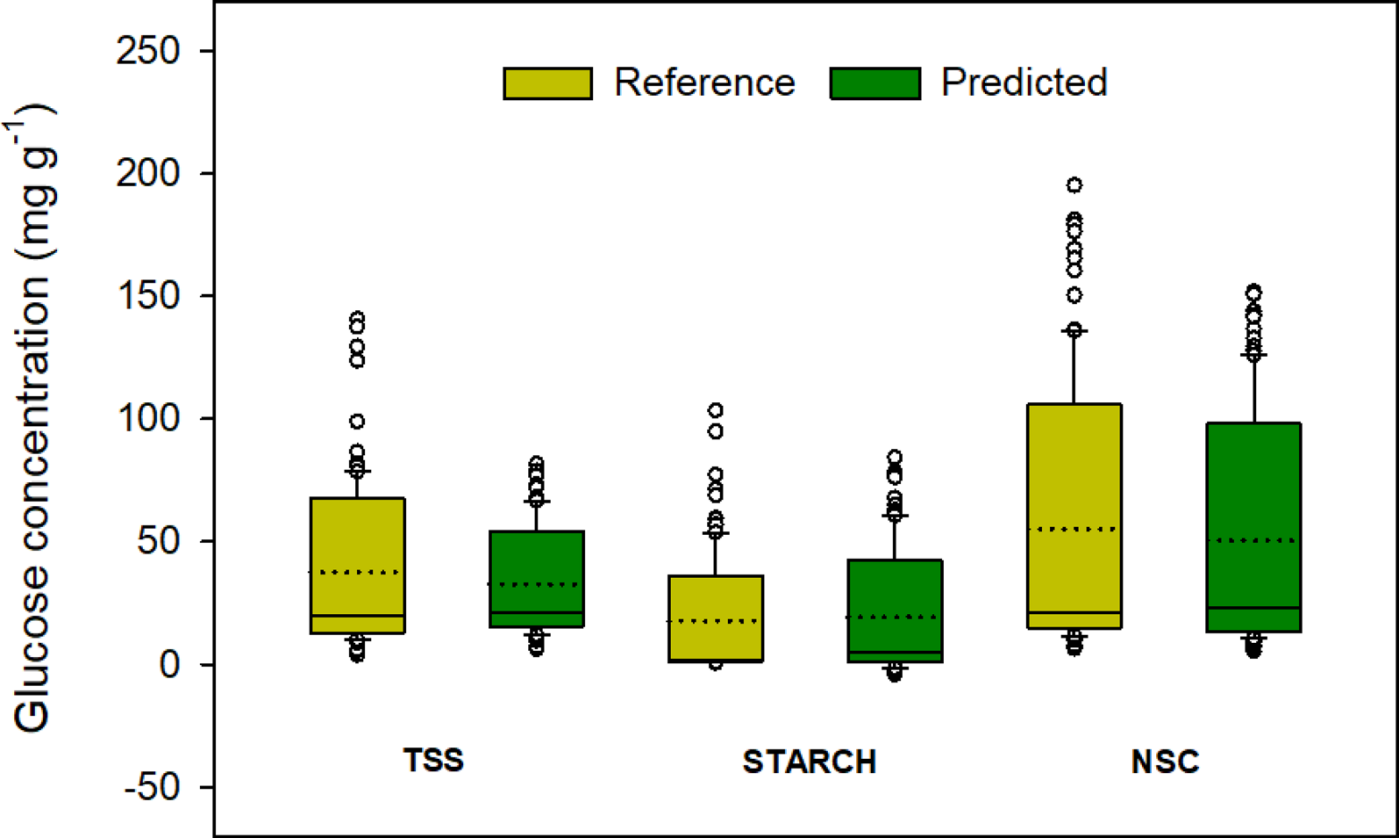
Visual representation of total soluble sugar (TSS), starch and non-structural carbohydrates (NSC) concentration (mg g^− 1^) dataset distribution based on reference (wet chemistry) and predicted values (FTIR) of 100 samples

The predicted values obtained from external validation were further examined. Here, the predictive models conservatively forecasted TSS, starch and NSC values (Fig. 5). For TSS PLS model, the difference of minimum and maximum values was 137.05 mg g^− 1^ in contrast to 75.63 mg g^− 1^ for predicted (Table 1). The pattern was similar with starch and NSC PLS models. The ranges of predicted values (starch = 88.64 mg g^− 1^, NSC = 146.54 mg g^− 1^) were also lower than the reference values (starch = 102.97 mg g^− 1^, NSC = 188.85 mg g^− 1^). As presented in Table 2, the mean and precision of reference TSS and NSC datasets were lower compared to the predicted values.

### Refinement of PLS regression models for TSS, starch and NSC concentration

**Fig. 6:**
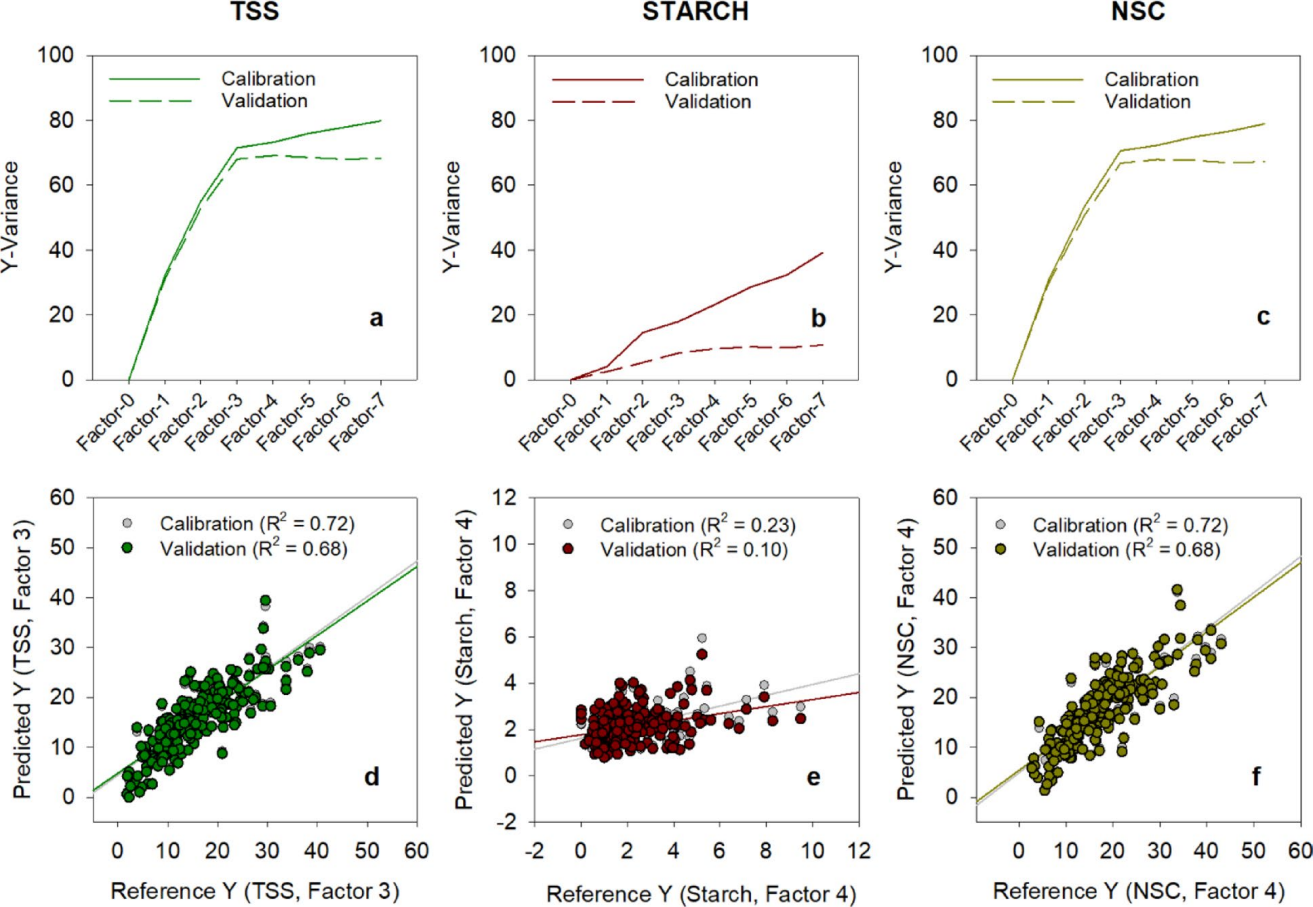
Overview of PLS models of rice leaves. Proportion of total variance in **a** TSS, **b** Starch, and **c** NSC concentrations accounted by the PLS model. PLS regression between reference (wet chemistry) and FTIR-predicted **d** TSS, **e** Starch, and **f** NSC concentrations

The enhanced transformation process of spectral datasets reduced noise and generated sharper peaks, highlighting the desired signal more distinctly. The PLS regression analysis of the leaf tissue models considered seven factors. The selected numbers of factors for TSS, starch and NSC are Factor-3, Factor-3 and Factor-4, respectively (Fig. 6a, b and c). Factor-1 contributed far more to the variability in the dataset compared to other factors. Across three parameters, all of the individual observations were concentrated around the center, suggesting a close correlation between Factor-1 and other selected factors. While majority of the data points were within the cluster, the TSS (Figure S5a) and starch (Figure S5c) models had more data points outside of the clustered data, compared with the NSC model (Figure S5e). These data points are outliers that are likely due to variability in physical properties of the leaf tissue samples or inadequate spectral preprocessing steps.

The association between latent variables and frequency exhibited clear strength and direction in the TSS (Figure S5b) and NSC (Figure S5f) models, with less noise compared to the starch model (Figure S5d). Among the chosen factors, the calibration model and internal validation demonstrated a more consistent percentage variation alignment with reference values, (Fig. 6d and e, and f). The alignment between the explained variance in reference content by the calibration model and predictions from internal validation was more pronounced in TSS and NSC models. In the three-factor calibration model, greatest percent accuracy was estimated in NSC (72%) and TSS (71%) with 68% of the variance in reference NSC and TSC contents for internal validation model. However, the starch model demonstrated a weaker ability to explain the variation in the reference values with 23% accuracy, and its internal validation accounted for a mere 9%.

The RMSE for both TSS and NSC models was approximately 4 mg g^-1^ for the calibration model and the internal validation (Table 3). In contrast, the RMSE values for the starch calibration model were 1.34 mg g^-1^ and 1.46 mg g^-1^ for the internal and external validation, respectively. These differences in the RMSE values were observed because the range of reference values was roughly the same for TSS and NSC and lower for starch (Table 1). Despite the starch model displaying lower RMSE, the R^2^ values (R^2^ = 0.23 for calibration model, R^2^ = 0.10 for internal validation) indicated that the model inadequately explained the variability in the reference starch content. In addition, the regression lines of the calibration model and internal validation were closely matched for the TSS (Fig. 6d) and NSC (Fig. 6f) models, but this alignment was not observed in the starch model (Fig. 6e) with MAPE values of calibration model and the internal validation for both TSS and NSC of 6% to 8%.

**Table 3:** Total soluble sugar (TSS), starch and non-structural carbohydrates (NSC) PLS models evaluation metrics using flag leaf tissues (*n* = 276)

Statistics	TSS	Starch	NSC
Principal components in models	3	4	4
R^2^ Training (*n* = 219)	0.73	0.23	0.72
R^2^ Internal Validation (*n* = 219)	0.69	0.10	0.68
R^2^ (External Validation using Model 1, *n* = 57)	0.28	–	0.16
R^2^ (External Validation using Model 2, *n* = 57)	0.63	–	0.66
RMSEC (Training)	4.09	1.34	4.24
RMSECV (Internal Validation)	4.35	1.46	4.57
RMSEP (External Validation using Model 1, *n* = 57)	5.06	4.06	5.58
RMSEP (External Validation using Model 2, *n* = 57)	3.62	1.18	3.55
RPD_C_ (Training)	1.9	1.1	1.9
RPD_CV_ (Internal Validation)	1.8	1.0	1.8
RPD_P_ (External Validation using Model 1, *n* = 57)	1.2	–	1.1
RPD_P_ (External Validation using Model 2, *n* = 57)	1.7	–	1.7
MAPE_C_ (Training)	7%	–	6%
MAPE_CV_ (Internal Validation)	8%	–	7%
MAPE_P_ (External Validation using Model 1, *n* = 57)	21%	–	4%
MAPE_P_ (External Validation using Model 2, *n* = 57)	5%	–	4%

### External validation of adjusted PLS regression models

**Fig. 7:**
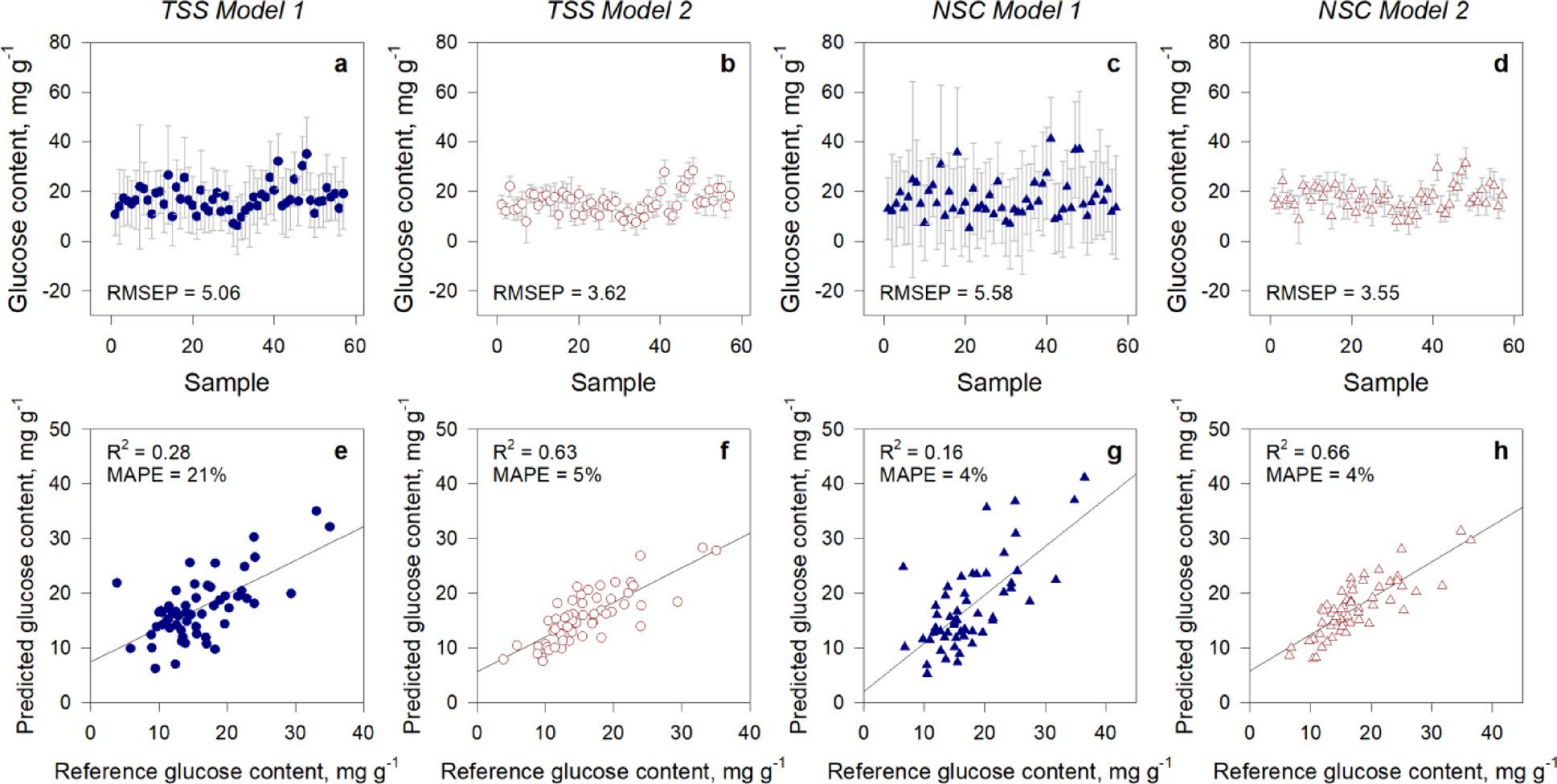
Comparison of TSS and NSC PLS regression models developed using both leaves and stem (Model 1) and leaves alone (Model 2) using different performance metrics. Deviation of individual data points and RMSEP of **a** TSS Model 1 and **b** TSS Model 2, **c** NSC Model 1, and **d** NSC Model 2. PLS regression between reference (wet chemistry) and predicted (FTIR) TSS and NSC content (mg g^− 1^) using different models: **e** TSS Model 1 and **f** TSS Model 2, **g** NSC Model 1, and **h** NSC Model 2

Two models were employed to predict TSS and NSC values. Model 1 utilized PLS regression using both leaves and stem samples while Model 2 was constructed using leaf samples only. The modification implemented in Model 1 led to a reduction in the root mean square error of prediction (RMSEP) by about 32% relative to the RMSEP of Model 1. For TSS models, RMSEP value was reduced from 5.06 mg g^-1^ (Model 1) to 3.62 mg g^-1^ (Model 2). Similarly, the RMSEP value of NSC models was lowered from 5.58 mg g^-1^ (Model 1) to 3.55 mg g^-1^ (Model 2). In comparison to Model 1 (Fig. 7a and c), the deviations of individual data points were reduced in the second model for both TSS and NSC (Fig. 7b and d). Here, the computed RMSEP using Model 2 streamlined the variability associated with the actual individual sample values.

The data predicted with Model 2, for both TSS and NSC, exhibited higher R^2^ values compared to R^2^ values of Model 1 (Table 3). The R^2^ value of TSS increased by 2.25-fold from 0.28 to 0.63 (Fig. 7e and g) while the R^2^ value of NSC increased by 4.1-fold from 0.16 to 0.66 (Fig. 7f and h). Our results demonstrated the Model 2 provided higher accuracy estimates of TSS and NSC than the TSS and NSC estimates using Model 1.

Computed RPD_P_ showed that Model 2 has a better predictive accuracy compared to Model 1 possibly because the test set (*n* = 57) are all leaves like Model 2. However, as shown by low RPD_P_ values from 1.1 to 1.7, the model may require further refinement by increasing the number of both training and test set. Furthermore, we have seen a 16% reduction in MAPE for TSS model while there was no change for NSC model using Model 2. Despite the significant difference observed in R^2^ and RMSE of the two models, MAPE was ineffective in gauging the enhancement in the model. This is because MAPE assigned equal importance to all errors, including those stemming from outliers. Consequently, substantial errors can disproportionately influence the overall calculation, diminishing the representativeness of MAPE in evaluating the overall model performance.

Altogether, enhancing the PLS regression model for TSS and NSC in rice tissues was achieved by employing homogenous samples and smoothening the spectra up to twenty-five points. This assessment further indicated that R^2^ and RMSE served as more precise metrics for evaluating the performance of the models than others.

### Data prediction using model 2

The response of different varieties under ambient (CTRL) and heated (HNT) conditions at nighttime were captured by the Model 2 (Figure S6a). The PLS regression Model 2 was used to predict the TSS and NSC contents in flag leaves of selected RDP 1 accessions (*n* = 30) for both cropping year 2019 and 2020 (Figure S6b).

**Fig. 8:**
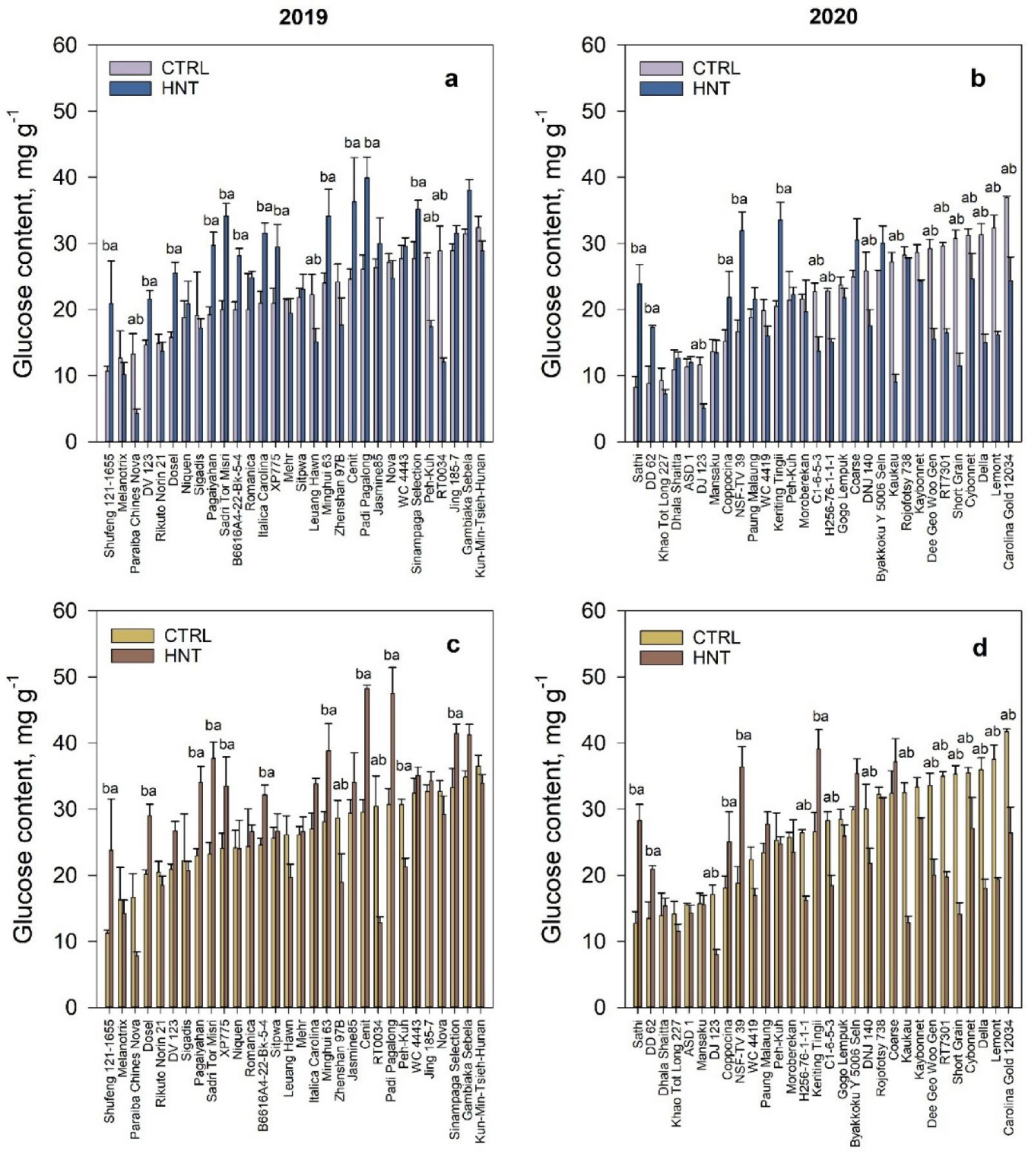
Total soluble sugar (TSS) and non-structural carbohydrates (NSC) in flag leaves of selected RDP1 rice accessions. Predicted TSS content in rice plants grown in cropping year **a** 2019 and **b** 2020. Predicted NSC content in rice plants grown in cropping year **c** 2019 and **d** 2020. All predicted values were quantified using the FTIR. Means with different letters above columns are significantly different between ambient (CTRL) and high nighttime temperature stress (HNT) treatment, according to Tukey’s Honest Significant Difference (HSD) test at α = 0.05

Rice stored various levels of TSS (Fig. 8a) and NSC (Fig. 8c) content in flag leaves after exposure to HNT stress during 2019. A total of 30 samples were chosen for both TSS and NSC prediction using Model 2 to assess the physiological response of rice crop to HNT stress (Table S1 and S2). Across all samples, TSS contents were highest in Padi Pagalong and lowest in Paraiba Chines Nova samples while NSC contents ranged from 7.8 to 48.2 mg g-^1^ predicted glucose content with greatest concentration measured in Cenit (Table S3 and S4). Due to their diverse population origins, research on these genotypes remains limited. Additionally, no studies have established a direct relationship between NSC content in their flag leaves and grain yield. Padi Pagalong, a member of the Tropical Japonica subpopulation, is known for its large sink size [49]. While Padi Pagalong’s average NSC content has not been documented in published literature, elevated TSS levels in the source may contribute to its sink capacity. Cenit, the genotype with the highest predicted NSC, is also classified within the Tropical Japonica subpopulation [50, 51], yet little research is available on its biochemical characteristics. This study estimated NSC content in selected genotypes from RDP 1 panel, demonstrating that FTIR spectroscopy can serve as an effective tool for assessing NSC levels in leaves, enabling characterization and screening of rare genotypes.

Among the 30 samples (Table S1), 47% exhibited no significant difference in TSS content of flag leaves under ambient and heated condition during 2019 cropping. In contrast, 40% of the overall samples displayed a substantial increase in TSS when grown inside the HNT tent, while only 13% demonstrated a significant decrease in TSS content. In the 2020 cropping, 53% of the rice varieties maintained consistent levels of NSC content in flag leaves while 33% showed a significant increase in NSC levels and the remaining 13% of the overall samples exhibited a significant decrease in NSC content under HNT stress (Table S2).

During the 2020 field experiment, the flag leaf samples from 30 rice accessions were used to assess both TSS (Fig. 8b) and NSC (Fig. 8d) prediction, mirroring the selection criteria from the previous year. Like 2019 growing season, roughly half of the selected accessions (43%) did not change TSS and NSC content of flag leaves following exposure to HNT stress. On the other hand, about 40% significantly reduced TSS and NSC content and 17% increased TSS and NSC content after imposition of HNT stress. Currently, no studies have explored the trend of NSC content in flag leaves among multiple varieties as influenced by HNT. However, in a recent study starch levels in the flag leaf of Nagina 22—a rice variety known for its tolerance to HNT—dropped by 87% under HNT conditions, with this decline occurring primarily during the latter portion of the night [52].

**Fig. 9:**
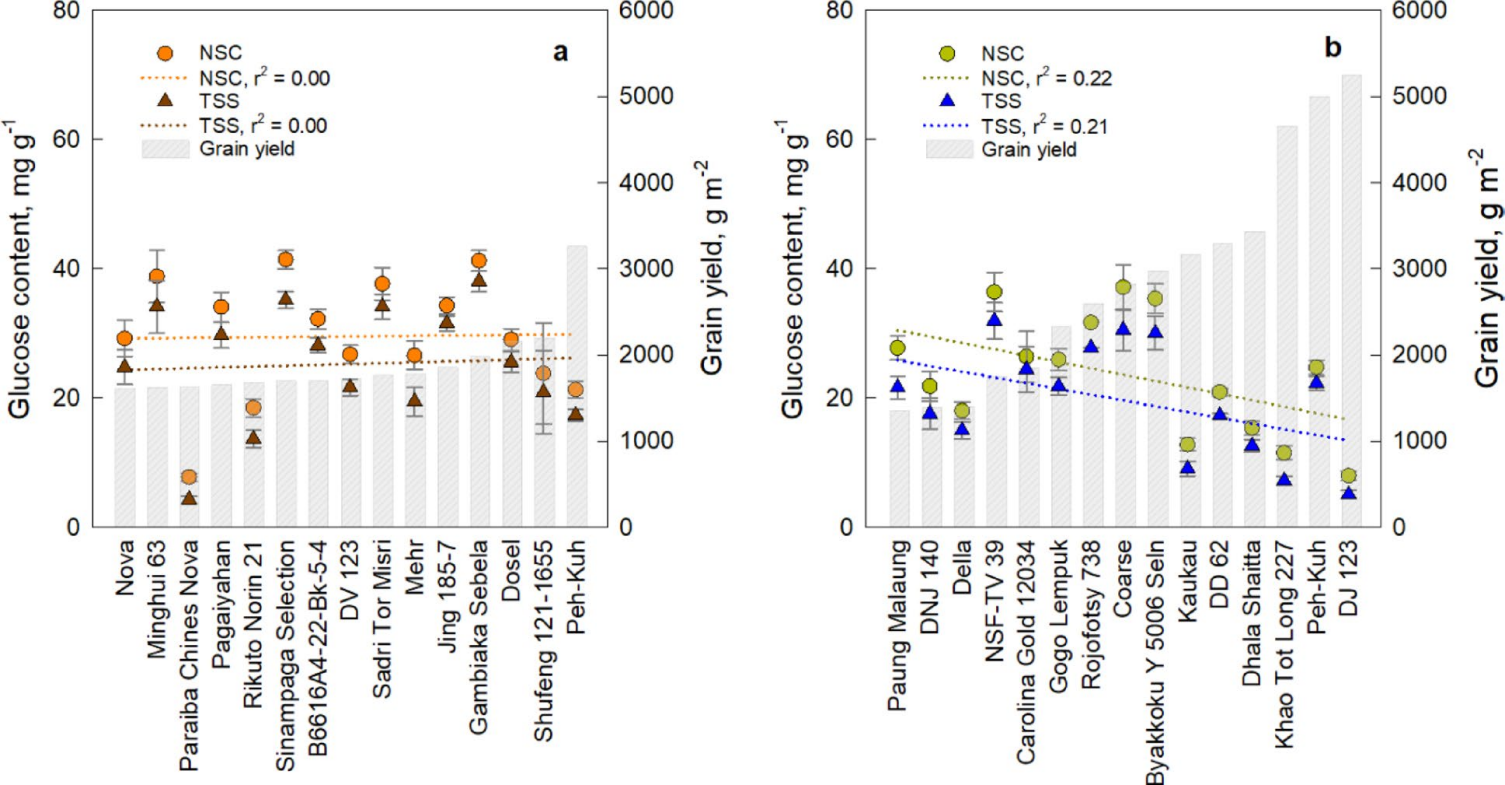
Total soluble sugar (TSS) and non-structural carbohydrates (NSC) in flag leaves of top 15 highest-yielding rice genotypes during cropping years **a** 2019 and **b** 2020. All predicted values were quantified using the FTIR. Bar graphs illustrate grain yield (g m^− 2^). The regression lines are represented by broken lines

In this study, the NSC content of the top 15 highest yield and most tolerant to HNT stress ranged from 7.8 to 41.5 mg g^− 1^ in 2019 and 8.0 to 37.1 mg g^− 1^ in 2020. In 2019, NSC and TSS levels did not show a consistent increase with yield (Fig. 9a). However, in 2020, a declining trend was observed for NSC (R² = 0.22) and TSS (R² = 0.21) as grain yield increased (Fig. 9b). Certain high-yielding genotypes under HNT stress during flowering exhibited lower NSC and TSS content, as predicted using Predictive Model 2. This suggests that, in several high-yielding rice varieties, assimilates may be translocated rapidly to the plant’s sink from the flag leaf, potentially enhancing tolerance to HNT stress.

### Comparative evaluation of PLS models for quantifying NSC in rice tissues

The outcomes of this study were assessed in relation to prior research with a shared objective of quantifying NSC in rice tissues, to elucidate discrepancies in model performance. Methodological parameters and key statistical indicators are summarized in Table 4. Notably, RMSE values were highest in the present study (3.55 to 19.1), whereas the lowest errors (0.025 to 0.02) were achieved using an NIR-based approach [17]. R² values from all studies—except Model 2 in this work—ranged between 0.86 and 0.96, demonstrating strong alignment between predicted and reference measurements. The notably low R² values observed in Model 2 are likely attributable to insufficient sample size.

**Table 4:** Comparison of PLS model performance for NSC quantification in rice tissues across related studies

	Wang et al., 2016 [17]	Xu et al., 2021 [16]	Mendez et al., this study	
			Model 1	Model 2
Tissue sample	Stem	Leaves and stem	Leaves and stem	Leaves
Total number of samples	434	526	565	276
Calibration Set	300	400	465	219
Validation Set	134	126	100	57
Spectroscopy	NIR	FTIR	FTIR	FTIR
Modelling software or package	R packages (signal, prospectr, pls)	R 3.6.2 (PLS model)	Unscrambler X 10.5.1	Unscrambler X 10.5.1
Spectral preprocessing	Standard normal variate, 1st derivative Savitzky-Golay	Standard normal variate, Savitzky-Golay	Savitzky-Golay, Baseline Transform, 2nd Derivative Savitzky-Golay, and Standard Normal Variate	Savitzky-Golay, Baseline Transform, 2nd Derivative Savitzky-Golay, and Standard Normal Variate
Subset selection	Kennard-Stone algorithm	Kennard-Stone algorithm	Kennard-Stone algorithm	Kennard-Stone algorithm
Principal components in models	8	13	4	4
R^2^ calibration	0.96	0.93	0.90	0.72
R^2^ internal validation	0.95	0.86	0.88	0.68
R^2^ external validation	0.92	0.90	0.92	0.66
RMSEC	0.02	1.475	17.6	4.24
RMSECV	0.02	2.066	19.1	4.57
RMSEP	0.025	1.559	15.3	3.55

Total sample counts across the three studies varied from 276 to 565, with differing splits between calibration and validation sets. This study featured the smallest validation subset, which may have contributed to reduced predictive accuracy. The influence of sample size on PLS model performance has been previously documented [53]. Another key methodological distinction concerns the number of principal components employed. This study utilized only four components, whereas prior studies adopted higher dimensional models. In accordance with Xu et al. [16], optimal component selection should be guided by RMSE minimization to enhance model performance. Overall, while calibration across models was successful, external predictability varied, likely influenced by differences in sample diversity, spectral technique, and preprocessing protocols.

Quantifying NSC content in flag leaves as a method to rapidly assess HNT tolerance is not fully explored but the finding of this experiment reinforced earlier studies indicating significance of flag leaf NSC in conferring HNT resilience in some rice accessions. The collection and processing of leaf samples are more efficient, therefore NSC content in flag leaves as measured through FTIR spectroscopy, could potentially be a viable phenotype for identifying HNT-resilient varieties. Nevertheless, the chemometric approach employed in this study could be evaluated by creating a flag leaf-based PLS regression model to screen a diverse rice panel, such as the RDP1.

## Conclusions

Ten rice accessions investigated for with and without HNT stress were selected to test the sensitivity of the FTIR model of varying concentrations of NSC in various plant parts. Flag leaves were chosen for predictive NSC analysis due to the convenience of sampling and processing, which is a solid basis for a high-throughput phenotyping strategy.

PLS regression analyses best fit the multivariate statistical method for identifying suitable plant parts for NSC predictions. Two distinct models were created for TSS, starch and NSC that were tailored to a specific tissue type. The distinct separation of latent variables when categorized by tissue type indicated that the spectra of leaf and stem tissues were distinguishable. Model 2 demonstrated improved accuracy in prediction, evident from its lower deviation and RMSEP compared to Model 1. However, it faced the challenge of predicting starch content in flag leaves due to the limited number of samples and the absence of values in the upper range of the regression. Therefore, expanding the population of the training set and incorporating samples characterized by high starch content are needed to improved Model 2.

Using PLS regression Model 2, roughly half of the population exhibited no significant difference in the NSC and TSS contents under ambient nor HNT stressed conditions for both 2019 and 2020 cropping seasons. The other half of the dataset, owing to its diversity, demonstrated a divergent response after the exposure to HNT stress. The PLS Model 2 was utilized to predict only 30 rice accessions from the entire RDP1 panel. Therefore, it is suggested that predicting the remaining 90% of the panel using FTIR spectroscopy could yield more conclusive results.

While flag leaf collection and processing are less intrusive, establishing improved PLS regression model for flag leaf TSS, starch and NSC can offer valuable insights. The approach employed in this study, particularly chemometric methodology, has the potential to enhance comprehension regarding RDP1 accessions, particularly in terms of their resilience to HNT.

## Supplementary Information

Below is the link to the electronic supplementary material.

Supplementary Material 1

Supplementary Material 2

## Data Availability

No datasets were generated or analysed during the current study.

## Abbreviations

ATR: Attenuated total reflectance
CTRL: Ambient
FTIR: Fourier transform infrared spectroscopy
HNT: High nighttime temperature (HNT)
IC: Ion chromatographic
IR: Infrared
LVS: Leaves
MAGIC^heat^: Multi-parent advanced generation inter-cross developed for heat tolerance
MAPE: Mean absolute percentage error
MAPE_C_: Mean absolute percentage error of calibration
MAPE_CV_: Mean absolute percentage error of cross validation
MAPE_P_: Mean absolute percentage error of prediction
MAX: Maximum value
MIN: Minimum value
MIR: Mid-infrared
NIR: Near-infrared
NSC: Non-structural carbohydrates
PLS: Partial least squares
R^2^: Coefficient of determination
RCDB: Randomized complete block design
RDP1: Rice diversity panel 1
RMSE: Root mean square error
RMSEC: Root mean square error of calibration
RMSECV: Root mean square error of cross validation
RMSEP: Root mean square error of prediction
RPD: Residual prediction deviation
RPD_C_: Residual prediction deviation of calibration
RPD_CV_: Residual prediction deviation of cross validation
RPD_P_: Residual prediction deviation of prediction
STD DEV: Standard deviation
STD ERROR: Standard error
TSS: Total soluble sugar
